# Cocaine-Triggered PR3-ANCA Vasculitis Localized to a Post-Surgical Neck Field: A Case of Locus Minoris Resistentiae in Drug-Induced Autoimmunity

**DOI:** 10.3390/diagnostics15161999

**Published:** 2025-08-10

**Authors:** Marko Tarle, Koraljka Hat, Lea Šalamon, Joško Mitrović, Marina Raguž, Danko Müller, Ivica Lukšić

**Affiliations:** 1 Department of Maxillofacial and Oral Surgery, Dubrava University Hospital, 10000 Zagreb, Croatia; tarlemarko1@gmail.com (M.T.); koraljkahat@gmail.com (K.H.); 2 School of Dental Medicine, University of Zagreb, 10000 Zagreb, Croatia; 3 Division of Clinical Immunology, Allergology and Rheumatology, Department of Internal Medicine, Dubrava University Hospital, 10000 Zagreb, Croatia; salamonlea@yahoo.com (L.Š.); jmitrovi@kbd.hr (J.M.); 4 School of Medicine, University of Zagreb, 10000 Zagreb, Croatia; danko.mueller@yahoo.com; 5 Faculty of Pharmacy and Biochemistry, University of Zagreb, 10000 Zagreb, Croatia; 6 Department of Neurosurgery, Dubrava University Hospital, 10000 Zagreb, Croatia; marinaraguz@gmail.com; 7 School of Medicine, Catholic University of Croatia, 10000 Zagreb, Croatia; 8 Department of Pathology and Cytology, Dubrava University Hospital, 10000 Zagreb, Croatia

**Keywords:** granulomatosis with polyangiitis, cocaine-induced vasculitis, PR3-ANCA, melanoma recurrence mimicry, neck dissection complications

## Abstract

**Background and Clinical Significance:** Cocaine-induced vasculitis (CIV), especially when associated with PR3-ANCA positivity, can be very similar both clinically and serologically to idiopathic granulomatosis with polyangiitis (GPA). The distinction between these entities is crucial due to the different etiologies, treatment strategies, and prognoses. We present a unique case of CIV that manifested exclusively in a previously dissected neck area—an example of the locus minoris resistance phenomenon—and was initially misinterpreted as skin melanoma recurrence. **Case presentation:** A 59-year-old man with a history of skin melanoma (pT4b, left pectoral region) and a previous modified radical neck dissection presented in 2024 with new onset of painful subcutaneous nodules and ulcerative lesions at the surgical site. The imaging procedures (CT and PET-CT) raised the suspicion of locoregional malignant recurrence. However, histology revealed necrotizing granulomatous inflammation without tumor cells. Extensive infectious and autoimmune investigations ruled out alternative causes. Subsequently, the patient developed a perforation of the nasal septum and ulcers on the oral mucosa. PR3-ANCA was strongly positive (up to 49 U/mL). Urine toxicology revealed intranasal cocaine use. A diagnosis of cocaine-induced PR3-ANCA vasculitis was made. After immunosuppressive therapy (high-dose glucocorticoids and methotrexate) and substance withdrawal counseling, the patient showed significant clinical improvement. **Conclusions:** This case highlights the importance of including CIV in the differential diagnosis of granulomatous or ulcerative lesions, especially when they are localized to previous surgical sites. The presentation illustrates the concept of locus minoris resistentiae and highlights the role of toxicological testing in atypical ANCA-positive disease.

## 1. Introduction

Granulomatosis with polyangiitis (GPA, formerly Wegener’s granulomatosis) is a rare anti-neutrophil cytoplasmic antibody (ANCA)-associated vasculitis with necrotizing and granulomatous inflammation of small vessels [1]. It belongs to the group of ANCA-associated vasculitides (AAV), together with microscopic polyangiitis and eosinophilic granulomatosis with polyangiitis. Cytoplasmic ANCA (c-ANCA) directed against proteinase-3 (PR3) predominates in GPA, while antibodies against myeloperoxidase (p-ANCA/MPO) are more common in other members of the group [2]. Clinically, GPA often presents with upper airway involvement (chronic sinusitis and/or nasal mucosal ulceration), pulmonary nodules and/or cavitary lesions, and rapidly progressive glomerulonephritis as a classic triad [1,3]. The clinical spectrum of GPA ranges from localized to life-threatening multisystem involvement [4,5]. ANCA and neutrophils are central to the pathogenesis of GPA, in which immune dysregulation and the formation of neutrophil extracellular traps (NETs) contribute to necrotizing inflammation and tissue damage [6,7,8,9,10]. However, due to its diverse manifestations and overlap with infections or malignancies, it remains a diagnostic challenge [1,3,11].

Cocaine-induced vasculitis (CIV), particularly cocaine adulterated with levamisole, has recently been recognized as an important clinical mimic of GPA [12,13]. Cocaine and levamisole induce strong neutrophil activation and NET formation, leading to the development of atypical ANCA autoantibodies, which perpetuate small-vessel inflammation and tissue damage [8,14,15]. Patients often have destructive upper airway lesions (chronic rhinitis, perforation of the nasal septum, and sinusitis) with systemic manifestations such as cutaneous vasculitis (purpura and ulceration), alveolar hemorrhage, and glomerulonephritis. In contrast to primary GPA, the serologic indication of a cocaine/levamisole etiology is the simultaneous presence of both ANCA types—e.g., high titers of PR3 (c-ANCA) and perinuclear ANCA with MPO or anti-HNE antibodies—a pattern that is very rare in idiopathic autoimmune vasculitis. ANCA positivity is found in over 90% of these patients, most commonly PR3 ANCA with an atypical p-ANCA pattern or dual PR3 and MPO positivity, whereas isolated MPO ANCA or ANCA negativity is less common in CIV [16]. Recognizing this etiology is extremely important as the therapeutic approach requires elimination of cocaine exposure with immunosuppression rather than a prolonged search for an underlying systemic vasculitic or autoimmune disease that is not present.

In patients with a previous oncologic diagnosis, particularly skin melanoma, the appearance of new ulcerative or granulomatous lesions in the area of a previous surgical procedure routinely raises suspicion of disease recurrence. However, granulomas are not specific to malignancy—sarcoid reactions are known to occur spontaneously or under the influence of immunotherapy and may mimic metastatic disease clinically and radiologically [17]. In addition, granulomatous vasculitides such as GPA and its drug-induced variants frequently occur in the head and neck region, and their masses may mimic neoplasms. Cases of GPA misinterpreted as sinus carcinoma or lymphoma, as well as cocaine-induced destructive lesions in the midline resembling infiltrative tumors, have been described in the literature [11].

## 2. Case Presentation

A 59-year-old man with a previous diagnosis of skin melanoma in the left pectoral region (stage pT4b) was treated in 2014 with wide local excision and re-excision of the margins and sentinel lymph node biopsy, which confirmed metastatic involvement of two lymph nodes at level V on the left side of the neck. A modified radical neck dissection was then performed. The patient underwent regular oncological monitoring until 2019, after which he was no longer monitored.

In September 2024, he presented with the appearance of multiple painful subcutaneous nodules and ulceronecrotic lesions at the site of the previous surgical procedure, accompanied by subfebrile fever, night sweats, and unintentional weight loss. Some of the lesions were inflammatory with purulent secretion. The microbiological smears of the wounds were mostly sterile. Staphylococcus epidermidis was isolated in one wound, but this was considered to be skin contamination. Due to a known allergy to penicillin, clindamycin and then trimethoprim/sulfamethoxazole were prescribed for treatment, but without a clinical response. A CT of the neck (November 2024) showed several changes suggestive of metastatic lesions: a marginally perfused subcutaneous mass in the left supraclavicular region (17 mm), a 10 mm lesion in the left submandibular area where the submandibular gland had previously been removed, and two perfused ovoid zones in the tail of the left parotid gland (16 and 14 mm). A similar lesion with a diameter of 11 mm was visible in the right tail of the parotid gland. A CT scan of the thorax, abdomen, and pelvis was also performed, which revealed no pathological findings. PET-CT (December 2024) showed multiple hypermetabolic foci from the mandibular angle to the supraclavicular region, including the left parotid gland, further strengthening the suspicion of a malignant etiology. Cytologic analysis of several lesions showed granulomatous inflammation with multinucleated giant cells, with no clear signs of malignancy. A right-sided cervical lymph node in level III showed cells suspicious for melanoma. The multidisciplinary tumor board recommended obtaining additional tissue samples and performing BRAF mutation analysis. The patient then underwent several biopsies—excisions and needle biopsies—of the neck and parotid gland lesions, all of which showed chronic inflammation and granulomatous changes, with no tumor cells found.

**Figure 1:**
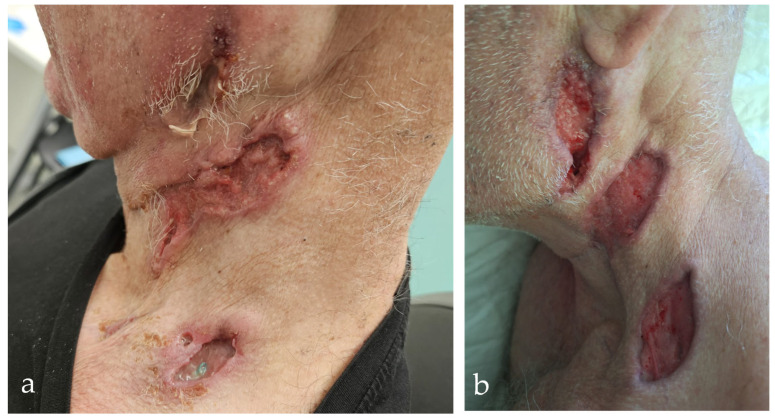
Ulcerative neck lesions before and after treatment in cocaine-induced PR3-ANCA vasculitis (CIV). (**a**) Initial presentation shows multiple necrotic–ulcerative skin lesions along the left mandibular border and in the area of the neck dissection, with erythema and induration. (**b**) Partial healing after glucocorticoids and antibiotic therapy, with granulation tissue and re-epithelialisation indicating a therapeutic response.

**Figure 2:**
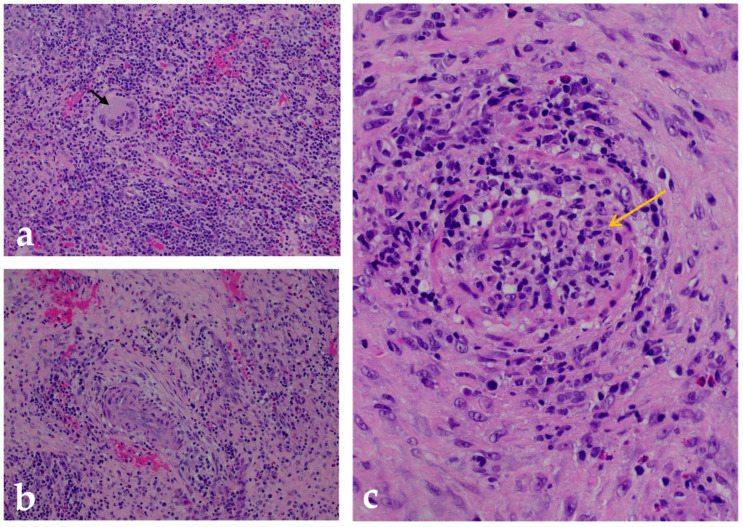
Histopathologic analysis of a GPA-associated cervical lesion. Hematoxylin and eosin (H&E)-stained sections from a biopsy of an ulcerative skin and subcutaneous lesion on the neck show the main histologic features of granulomatosis with polyangiitis (GPA): (**a**) A well-formed necrotizing granuloma embedded in a fibrotic stroma surrounded by a dense mixed inflammatory infiltrate composed predominantly of lymphocytes and neutrophils. In the center are multinucleated Langhans-type giant cells (black arrow), which are characteristic of GPA-related granulomatous inflammation. Magnification: 200×. (**b**) Perivascular inflammation involving small caliber vessels within fibrotic connective tissue. It shows segmental wall necrosis and a dense infiltrate of lymphocytes and neutrophils surrounding the affected vessels—findings consistent with chronic vasculitic changes. Magnification: 200×. (**c**) High magnification (400×) of a small- to medium-caliber arteriole with features of necrotizing vasculitis, including transmural infiltration of inflammatory cells (yellow arrow) and extensive fibrinoid necrosis of the vessel wall—hallmarks of active GPA.

At the beginning of February 2025, the patient was admitted to the Department of Maxillofacial Surgery due to the clinical course and his poor general condition. Clinically, there were several confluent ulceronecrotic lesions on the left side of the neck, including a lesion on the tail of the parotid gland (2 cm), which secreted purulent contents when pressed. Two subcutaneous nodules were palpated in the left parotid gland: one nodule in the left supraclavicular fossa and an additional 1 cm ulceration in this region. The infraclavicular skin was edematous. No pathologic lymph nodes were palpated on the right side of the neck (Figure 1). The patient subjectively reported hearing loss in the left ear and occasional pain. There were clots and dried blood in both nostrils, which the patient attributed to mechanical trauma. Laboratory results showed inflammatory activation with a CRP of 77.2 mg/L (reference < 5 mg/L). The rest of the physical examination was unremarkable. In particular, there were no neurological deficits (no evidence of polyneuropathy), and renal function was within normal limits. The wound swab was sterile. Serologic findings showed positive PR3-ANCA (13 U/mL (reference < 5 U/mL) by ELISA, consistent with a cytoplasmic ANCA pattern) and borderline p-ANCA/MPO (5 U/mL (reference < 5 U/mL)), while ANA, anti-dsDNA, ENA, C3, and C4 were normal. At the same time, a comprehensive examination for infectious diseases was carried out to rule out infectious causes for the granulomatous and ulcerative lesions. Serological, microbiological, and histopathological tests were performed to rule out the most important infections—including tuberculosis, atypical mycobacteria, fungal diseases, syphilis, Bartonella henselae, toxoplasmosis, nocardiosis, actinomycosis, HIV, and hepatitis B and C—all of which could be excluded. Histopathological analysis of one cervical ulceration confirmed necrotizing granulomatous inflammation and small-vessel vasculitis with features highly suggestive of GPA (Figure 2). After ruling out infectious and malignant causes, we suspected systemic autoimmune vasculitis based on the clinical and laboratory findings. However, no immunosuppressive therapy was initiated during his hospitalization, and the patient was discharged with a recommendation for further diagnostic evaluation and monitoring.

**Figure 3:**
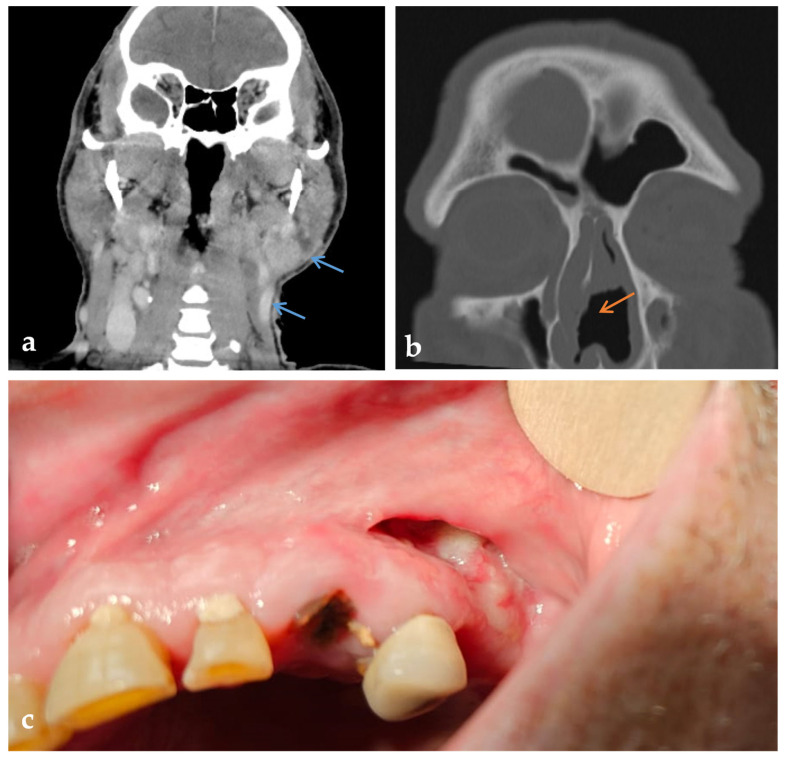
Radiologic and intraoral findings in PR3-ANCA-positive cocaine-induced vasculitis (CIV). (**a**) Coronal contrast-enhanced CT showing soft tissue lesions with increased enhancement in the left supraclavicular and parotid region (blue arrows). (**b**) Axial CT of the paranasal sinuses with perforation of the nasal septum (orange arrow). (**c**) Intraoral view showing mucosal ulceration and exposed bone in the upper left oral vestibule.

In April 2025, he was hospitalized again due to the deterioration of the local findings and his general condition. The ulcerations became larger, deeper, and confluent, turning into a wide wound with purulent contents and signs of acute infection. The CRP level was 226 mg/L, and the hemoglobin level was 103 g/L (reference 138–175 g/L). A newly developed perforation of the nasal septum with a diameter of about 2 cm was discovered as well as an ulceration in the area of the upper vestibular region on the left side of the oral cavity (Figure 3). A subsequent CT scan of the head, neck, and thorax showed progression—the submandibular lesion enlarged to 32 mm (previously 10 mm) with invasion of the skin at the level of the hyoid bone and progressive thickening of the mucosa of the oral cavity with extension into the nasopharynx and oropharynx (Figure 3). The wound swabs remained sterile, and Streptococcus mitis/oralis was isolated from the ulcer in the oral cavity, which was interpreted as a commensal bacterium. Urine toxicology was positive for cocaine, which the patient subsequently confirmed—with chronic intranasal use. Bronchoscopy showed no evidence of trachea, bronchi, or lung involvement. BAL was negative for infection, and pigmented macrophages were present. Cardiac ultrasound showed no signs of endocarditis. Systemic therapy with methylprednisolone (0.5 mg/kg) and a combination of meropenem and vancomycin for suspected concomitant bacterial infection was initiated. The therapy led to an improvement in the local findings and a decrease in the inflammatory parameters (Figure 1). The day before discharge, the patient experienced an episode of vomiting and drowsiness, which resolved after symptomatic treatment. The interview confirmed acute benzodiazepine intoxication. The patient was discharged hemodynamically stable, afebrile, and in an improved clinical condition. The PR3-ANCA level at this time was 49 U/mL (reference < 5 U/mL).

**Figure 4:**
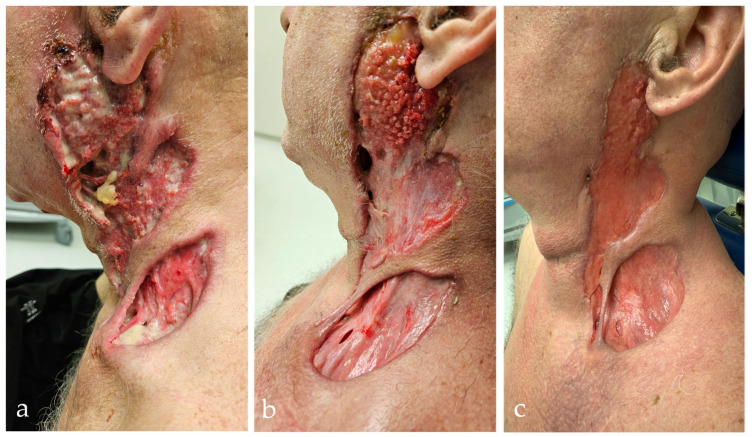
Worsening and response to therapy in PR3-ANCA-positive vasculitis during the third hospitalization. (**a**) Extensive ulcerative–necrotic lesions on the left side of the neck with infiltration of the parotid gland region. The lesions involve deep layers of subcutaneous and muscle tissue with clear signs of inflammation. (**b**) Partial improvement after systemic corticosteroid therapy, with visible granulation tissue, reduced necrosis, and early re-epithelialization. (**c**) Further epithelialization and regression of inflammation after three weekly doses of subcutaneous methotrexate and daily oral methylprednisolone.

A third hospitalization followed in May 2025 due to the recurrence of ulcerations and the development of paresis of the marginal branch of the left facial nerve. Pulse therapy with methylprednisolone (500 mg i.v. over 3 days), continued with methylprednisolone per os (64 mg/day) and methotrexate 15 mg s.c. once a week, was initiated. Antimicrobial therapy with vancomycin and meropenem was administered for 12 days. The therapy led to epithelialization of the lesions, reduced discharge, a decrease in CRP, and a regression of inflammatory parameters (Figure 4). The liver enzymes showed a temporary increase without the therapy having to be interrupted. The wound swab remained sterile. The patient was discharged in good general condition, fever-free, and without subjective complaints. The treatment plan includes the continuation of methotrexate and a gradual reduction in the glucocorticoid dose with multidisciplinary monitoring and toxicologic control due to the previously documented cocaine dependence. During hospitalizations, the patient repeatedly denied cocaine use. However, not every toxicology urine test was positive for this substance. After drug use was confirmed, the patient was under regular psychiatric observation.

## 3. Discussion

This case shows a rare manifestation of CIV occurring at the site of a previous surgical procedure, namely a neck dissection. Such localization is consistent with the concept of locus minoris resistentiae, which refers to areas of prior structural or immunological impairment that become more susceptible to subsequent pathological processes. In this patient, the surgically altered neck region likely acted as a point of reduced resistance where vascular inflammation became clinically apparent after cocaine exposure. Recognizing this pattern may help clinicians identify the underlying etiology more quickly and avoid unnecessary diagnostic delays.

The patient presented with PR3-ANCA-positive cocaine-induced vasculitis, which clinically and serologically resembled idiopathic granulomatosis with polyangiitis (GPA). Cocaine abuse (especially when adulterated with levamisole) is recognized as a trigger for ANCA-associated small-vessel vasculitis, with manifestations ranging from localized destructive lesions—most commonly in the midline, also known as cocaine-induced midline destructive lesions (CIMDL)—to systemic disease [8,17]. The most frequent clinical features of CIV include sinonasal involvement, retiform purpura or necrotic skin ulcers, and systemic symptoms such as fever, arthralgias, or weight loss. In more severe cases, pulmonary hemorrhage or pauci-immune glomerulonephritis may occur, while isolated neurological or renal complications have also been described. However, upper respiratory and cutaneous involvement predominate, particularly in patients who actively use levamisole-adulterated cocaine [8]. Pathogenetically, cocaine and levamisole induce strong neutrophil activation and NETosis; it has been shown in vitro that ANCA antibodies associated with cocaine exposure can induce primed neutrophil granulocytes to undergo a respiratory burst and degranulation with the formation of NETs [8]. These NETs are particularly rich in neutrophil elastase (HNE) and other enzymes that promote loss of tolerance to self-antigens and favor the development of atypical ANCAs that target both MPO/PR3 and HNE. As a result, autoantibodies (c-ANCA, p-ANCA, and anti-HNE) develop and persist, further activating neutrophils and initiating a vicious cycle of chronic inflammation and small-vessel damage [10,14,15,18]. Clinically, CIV is difficult to distinguish from primary GPA—patients may present with destructive upper airway lesions, pulmonary infiltrates, skin ulcers, and positive ANCA findings. However, certain features may indicate a secondary etiology [12,19,20]. In cocaine vasculitis, “double” ANCA positivity or atypical specificities are common: for example, the simultaneous presence of PR3-ANCA (c-ANCA) and MPO-ANCA (p-ANCA) as well as antibodies against human neutrophil elastase (HNE). Such a combined pattern is extremely rare in idiopathic GPA [21]. In our case, during all phases of disease exacerbation, PR3-ANCA levels were continuously elevated, while MPO-ANCA levels remained within the reference range or at the upper limit of the normal range. As no anti-HNE antibody test was available in our institutional laboratory, we were unable to examine this marker, which has been associated with CIV in previous studies. In recent series of cocaine-induced vasculitis, more than half of the patients have PR3-ANCA, while MPO-ANCAs are very rare or absent. In addition, anti-HNE antibodies are detected in a large proportion of patients with CIMDL (in 28–84% of cases), whereas in primary GPA such “atypical” ANCAs are rare [22]. Despite these differences, the serologic profile alone is not sufficient to reliably distinguish idiopathic from drug-induced disease. The clinical course and distribution of the lesions may provide additional clues: idiopathic GPA often affects multiple organ systems (e.g., lungs and kidneys) and has a chronic, progressive course, whereas CIV may be limited to skin and ENT manifestations that occasionally improve after cessation of cocaine exposure [23]. A detailed medical history and toxicologic analysis play an important role in the differential diagnosis. As patients often do not report cocaine use, active urine testing is recommended for any atypical form of vasculitis [24]. In a recent study, up to 32% of patients with cocaine vasculitis denied cocaine use, resulting in a positive toxicology finding [12]. In patients with destructive lesions clinically resembling GPA, cocaine use should be excluded as a precipitating cause in all cases before initiating aggressive immunosuppressive therapy [25]. Furthermore, the intensity of any immunosuppressive treatment should be guided by the severity of the clinical presentation rather than by the underlying trigger. The special feature of this case is the phenomenon of locus minoris resistentiae—the vasculitis was localized exclusively in the area of a previous surgical procedure on the neck. The surgical scar and surrounding tissue represent an “immunologically compromised area” in which tissue damage and impaired innervation or lymphatic drainage alter the local immunological response [26].

Locus minoris resistentiae is defined as a site that is more susceptible to disease than the surrounding healthy tissue. Cases of granulomatous inflammation and vasculitis confined to previous scars have been described in the literature—for example, the reactivation of eosinophilic granulomatosis with polyangiitis (Churg–Strauss) within old surgical scars [27]. It is assumed that permanent microstructural changes (fibrosis, devascularization, and altered local immunity) create a favorable environment for a focal autoimmune reaction [28]. In our case, it was the operated area of the neck—which had been the site of lymphatic dissection and scarring for years—that became the target of cocaine-induced destructive vasculitis. This unusual localization further complicated the recognition of the disease: the initial appearance of ulcerations in the area of a previous melanoma was interpreted as a possible local recurrence of the malignancy. Granulomatous inflammation detected in biopsies of lesions was an equivocal finding—granulomas may accompany not only malignant tumors (sarcoid reaction) or infections but also systemic autoimmune diseases [29,30,31]. Cases have been described in which GPA was mistaken for a malignancy of the paranasal sinuses or nasopharynx, as well as cases of destructive cocaine-induced nasal lesions mimicking invasive tumors [24,32]. Therefore, it was important to examine the tissue samples microscopically and microbiologically and to perform a serologic examination in parallel. In our patient, several biopsies (cervical ulcerations and nodules in the parotid gland region) were performed, which consistently showed necrotizing granulomas with vasculitis, but without tumor cells or infectious agents. Such finding, together with the positivity of PR3-ANCA, suggested a diagnosis of GPA-related disease. However, only the toxicologic analysis (positive test for cocaine) resolved the dilemma between idiopathic and induced vasculitis. In our case, cocaine use was confirmed by a positive urine test, and the patient reported regular intranasal use for over 10 years. Hair testing was not feasible due to baldness, and additional testing was not considered necessary given the clear history and laboratory confirmation. It should be noted here that levamisole, a common adulterant of cocaine, is an additional factor in the pathogenesis—up to 70–80% of illicit cocaine samples contain levamisole, which in turn can cause autoimmunity [33,34]. Ultimately, determining the exact etiology is crucial for treatment: primary GPA requires long-term immunosuppression, whereas in CIV the therapeutic strategy is primarily aimed at interrupting the causative pathogen. Unfortunately, detection of levamisole in biological samples was not available at our institution, and we were therefore unable to confirm its presence as a potential co-factor in the pathogenesis of vasculitis. The literature emphasizes that cessation of cocaine use is an important prerequisite for remission of the disease—without this, immunosuppressive therapy alone often does not lead to healing of the lesions [8,12]. In our case, once the diagnosis was confirmed, the patient was started on high-dose glucocorticoid treatment with methotrexate, with strict instructions to abstain from cocaine. With such a multidisciplinary approach (surgical care, immunologic treatment, and psychiatric support for withdrawal), gradual healing of the ulcerations was achieved. This review highlights several important lessons for clinicians. First, the possibility of drug-induced vasculitis should always be considered in the differential diagnosis of atypical ulcerative–destructive lesions, even in patients without an obvious history of addiction. Secondly, new lesions at the site of a previous surgical procedure in patients with a history of oncology do not always mean recurrence of the disease—it is necessary to confirm the diagnosis histopathologically and consider other causes (infections, sarcoidosis, and vasculitis). Thirdly, Clinicians should suspect a drug-induced vasculitis when patients present with red-flag features like unexplained destructive midline nasal lesions or widespread purpuric skin lesions, particularly if serologic testing reveals atypical patterns (for example, concurrent positivity for both PR3-ANCA and MPO-ANCA). Notably, because patients may deny cocaine use, confirmatory toxicology screening (e.g., a urine cocaine test) is advisable in such suspicious cases—even if toxicology tests are not part of the routine vasculitis workup—to ensure that the correct diagnosis is not missed. Instead of unnecessary aggressive oncologic or immunosuppressive therapy, the first step is to eliminate exposure to cocaine. This is followed by targeted immunosuppressive treatment of the more severe manifestations according to the principles for AAV, but with careful consideration of the benefits and risks [35]. Ultimately, successful treatment of such patients requires the collaboration of multiple specialists—from ENT and maxillofacial surgeons, pathologists, and psychiatrists to rheumatologists, infectious disease specialists, and toxicologists—to ensure a correct diagnosis and comprehensive treatment.

## 4. Conclusions

Cocaine-induced vasculitis (CIV) may mimic malignancy or autoimmune vasculitis, particularly when it presents as necrotizing granulomatous lesions. To our knowledge, this is the first reported case of PR3-ANCA-positive CIV strictly limited to a previous surgical site, supporting the concept of locus minoris resistentiae. CIV should be considered in the differential diagnosis of destructive lesions, even if drug use is denied, and targeted toxicological testing is essential. Cessation of cocaine use remains the cornerstone of treatment, while immunosuppressive therapy should be tailored to the severity of the disease. Early detection and individualized management are key to a favorable outcome.

## Data Availability

All data used are available within this article.

## Abbreviations

The following abbreviations are used in this manuscript:

ACE	Angiotensin-Converting Enzyme
AAV	ANCA-Associated Vasculitis
ANA	Antinuclear Antibody
ANCA	Anti-Neutrophil Cytoplasmic Antibody
anti-CCP	Anti-Cyclic Citrullinated Peptide Antibody
anti-dsDNA	Anti-Double-Stranded Deoxyribonucleic Acid Antibody
anti-HNE	Anti-Human Neutrophil Elastase Antibody
BAL	Bronchoalveolar Lavage
CIMDL	Cocaine-Induced Midline Destructive Lesions
CIV	Cocaine-Induced Vasculitis
CRP	C-Reactive Protein
GPA	Granulomatosis with Polyangiitis
HNE	Human Neutrophil Elastase
KOH	Potassium Hydroxide
MPO	Myeloperoxidase
MTX	Methotrexate
NETs	Neutrophil Extracellular Traps
p-ANCA	Perinuclear Anti-Neutrophil Cytoplasmic Antibody
PR3	Proteinase 3
RPR	Rapid Plasma Reagin
